# Microscopic polyangiitis with isolated cardiopulmonary involvement: A case report

**DOI:** 10.1097/MD.0000000000044659

**Published:** 2025-09-19

**Authors:** Jing Chen, Lvjun Zhang, Zhengming Huang, Dan Zhu

**Affiliations:** a Department of Respiration and Critical Care Medicine, Affiliated Jinhua Hospital, Zhejiang University School of Medicine, Jinhua Municipal Central Hospital, Jinhua, Zhejiang Province, China.

**Keywords:** heart failure, interstitial pneumonia, microscopic polyangiitis, pleural effusion

## Abstract

**Rationale:**

Microscopic polyangiitis (MPA), a subtype of antineutrophil cytoplasmic antibody (ANCA)-associated vasculitis, is typically characterized by renal impairment but can present in atypical forms. This case underscores an unusual manifestation of MPA, involving solely cardiopulmonary pathology, thereby highlighting the necessity for increased clinical vigilance even in the absence of renal involvement.

**Patient concerns:**

An 87-year-old female patient with atrial fibrillation, hypertension, and type 2 diabetes presented with progressive chest tightness, bilateral limb and facial edema, and fatigue over 3 months. Initial evaluations suggested pneumonia accompanied by heart failure. However, standard treatment provided only temporary symptomatic relief.

**Diagnosis:**

Laboratory tests indicated that the level of myeloperoxidase ANCA reached 122 RU/mL. High-resolution computed tomography of the chest revealed characteristic findings of interstitial pneumonia, including thickening of lung lobular septa, bilateral ground-glass opacities, and pleural effusion, while other indicators did not suggest renal impairment. As a result, based on the 2022 American College of Rheumatology/European League Against Rheumatism standards, the final diagnosis was MPA.

**Interventions:**

The patient was given 80 mg of intravenous methylprednisolone daily for 3 days, then the dose was reduced to 60 mg daily for another 3 days. Afterwards, the patient was switched to oral prednisone 40 mg daily combined with 0.5 g of mycophenolate mofetil twice daily, and responded well to the treatment.

**Outcomes:**

On the 6th day of hospitalization, the patient was discharged without complications. Follow-up blood tests that conducted 3 weeks post-discharge indicated normal liver and kidney function. A chest computed tomography scan revealed substantial resolution of bilateral pleural effusion. The majority of ground-glass opacities had resolved, with only a few persisting, primarily in the peripheries of the lobes.

**Lessons:**

This case suggests that MPA may present with isolated cardiopulmonary involvement as the initial manifestation without typical renal damage. Clinicians should consider MPA in patients with refractory interstitial pneumonia, pleural effusion, and myeloperoxidase ANCA positivity. Early immunosuppressive therapy may reverse organ damage and improve outcomes.

## 1. Introduction

Antineutrophil cytoplasmic antibody (ANCA)-associated vasculitis (AAV) is a rare disorder that predominantly affects small blood vessels across multiple organ systems. Microscopic polyangiitis (MPA) and granulomatosis with polyangiitis are subtypes of AAV. MPA is primarily associated with myeloperoxidase antineutrophil cytoplasmic antibody (MPO-ANCA), whereas granulomatosis with polyangiitis is mainly linked to proteinase 3 (PR3)-ANCA.^[1]^ Typically, MPA involves renal manifestations such as hematuria, proteinuria, and a rapid decline in renal function. However, we present an atypical case of MPA characterized by exclusive involvement of the heart and lungs, without renal involvement. The patient exhibited symptoms of interstitial pneumonia and heart failure. This case report aims to aid clinicians in the early identification of MPA.

## 2. Case presentation

An 87-year-old female patient with a medical history of atrial fibrillation, hypertension, and type 2 diabetes mellitus presented with the symptoms of chest discomfort, bilateral lower extremity and facial edema, and progressive weakness persisting over a 3-month period. Initial diagnostic evaluations indicated elevated levels of B-type natriuretic peptide (BNP), bilateral pleural effusion, and pulmonary inflammatory exudation as observed on chest computed tomography (CT). Despite these findings, the condition was diagnosed by other departments as pneumonia concomitant with heart failure. The administration of anti-infective therapy, cardiotonic agents, and diuretics resulted in temporary symptomatic relief; however, the patient’s condition subsequently deteriorated.

**Figure 1.**
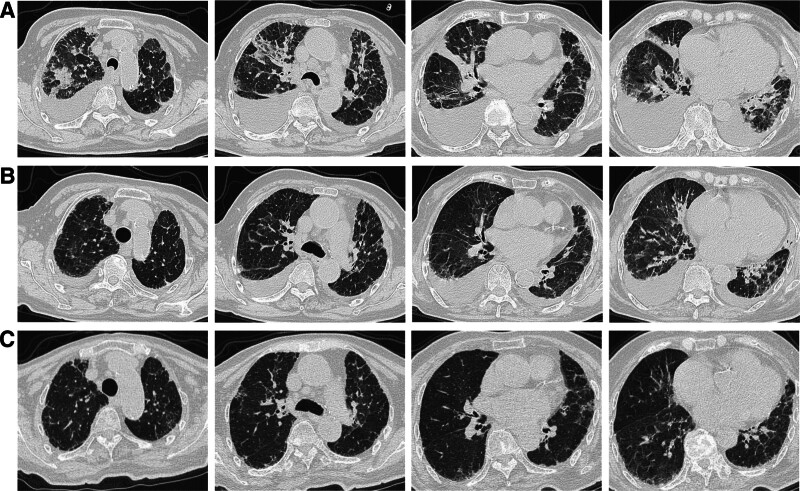
(A) Upon admission to our department, a chest CT examination of the patient revealed ground-glass opacities in both lungs, thickening of the interlobular septa, and bilateral pleural effusion. (B) A chest CT scan conducted after 6 days of treatment demonstrated improvement in both pulmonary conditions. (C) Three weeks post-discharge, the patient exhibited no evidence of bilateral pleural effusion, demonstrated significant absorption of ground-glass opacities in both lungs, and presented with a few reticular opacities beneath the pleura in both lungs. CT = computed tomography.

Finally, the patient was admitted to our department for comprehensive evaluation. Laboratory investigations revealed the following results: an antinuclear antibody titer of 1:160, MPO-ANCA level of 122, erythrocyte sedimentation rate of 38 mm/h, alanine aminotransferase level of 62.8 U/L, aspartate aminotransferase level of 70 U/L, lactate dehydrogenase level of 300 U/L, platelet count of 366 × 10^9^/L, immunoglobulin G level of 16.2 g/L, immunoglobulin G4 level of 2.29 g/L, and BNP level of 212 pg/mL, with renal function tests within normal parameters. Urinalysis yielded no significant findings, and other laboratory indicators were within normal limits. A CT scan of the chest revealed interstitial pneumonia, characterized by thickening of the interlobular septa, ground-glass opacities in both lungs, and bilateral pleural effusion (Fig. 1A). The echocardiogram revealed enlargement of both atria, moderate regurgitation of the mitral and tricuspid valves, mild to moderate regurgitation of the aortic valve, and arrhythmia.

An examination of the patient’s prior hospitalization records indicated a persistent elevation of MPO-ANCA levels. A thorough assessment was conducted by synthesizing clinical presentations, laboratory data, and thoracic imaging findings to arrive at a diagnosis. The patient demonstrated multisystem involvement, culminating in a diagnosis of MPA, despite the absence of renal impairment. Treatment was initiated with methylprednisolone at a dosage of 80 mg daily for 3 days, subsequently tapered to 60 mg daily for an additional 3 days. Notable improvement was observed in thoracic imaging after 6 days, accompanied by a marked alleviation of symptoms (Fig. 1B). The patient was discharged with a prescription for prednisone at 40 mg daily and mycophenolate mofetil at 0.5 g twice daily. At a follow-up appointment 3 weeks later, blood tests indicated normal hepatic and renal function. A chest CT scan demonstrated a significant resolution of the bilateral pleural effusion, with most ground-glass opacities resolved, except for a few predominantly located in the peripheral regions of both lobes (Fig. 1C).

## 3. Discussion

MPA is an autoimmune disorder characterized by its impact on various organs, notably the kidneys, lungs, and skin.^[2]^ A defining characteristic of MPA is necrotizing vasculitis. The clinical manifestations of MPA are highly variable, with nonspecific symptoms such as fever, weight loss, and weakness. Patients may present with either an acute onset or a gradual progression of symptoms prior to a definitive diagnosis.^[3]^ In the case under consideration, the patient primarily exhibited systemic debilitation, in the absence of fever or weight loss, and experienced a gradual progression of the disease.

In 2022, the American College of Rheumatology, in conjunction with the European Alliance of Associations for Rheumatology, introduced revised classification criteria for MPA.^[4]^ These criteria exhibit a sensitivity of 91% and a specificity of 94%. The classification criteria for MPA are that Nasal involvement: bloody discharge, ulcers, crusting, congestion, blockage, or septal defect/perforation (−3). Positive test for perinuclear antineutrophil cytoplasmic antibodies or antimyeloperoxidase (anti-MPO) antibodies ANCA positive (+6). Fibrosis or interstitial lung disease on chest imaging (+3). Pauci-immune glomerulonephritis on biopsy (+3). Positive test for cytoplasmic ANCA or antiproteinase3 (anti-PR3) antibodies (−1). Blood eosinophil count ≥1 × 10^9^/L (−4).^[4]^ According to these guidelines, a patient achieving a score of ≥5 points is eligible for an MPA diagnosis. It can be found from the above classification criteria that patients with AAV are classified as having MPA easier if the present with the following characteristics: absence of sino-nasal symptoms or signs, lack of cytoplasmic ANCA or PR3-ANCA positivity, and an eosinophil count below 1 × 10⁹/L. This criterion underscores that a patient may demonstrate perinuclear-ANCA/MPO-ACNA positivity in isolation, without the necessity of lung or kidney involvement. Consequently, lung and kidney involvement are not requisite for an MPA diagnosis. Our patient exhibited MPO-ANCA positivity (+6) and interstitial lung disease (+3), culminating in a total score of 9 points. Accordingly, we confidently classified her condition as MPA.

Certain studies suggest that MPO-ANCA-positive interstitial pneumonia can manifest independently of other organ involvement, a condition termed pulmonary-limited AAV.^[5]^ Additionally, there are documented cases of vasculitis presenting without renal involvement.^[6]^ These findings further support the notion that renal involvement is not a requisite criterion for the diagnosis of AAV. In the case under discussion, the patient exhibited no renal involvement but presented with heart failure, bilateral pleural effusion, and interstitial pneumonia. The patient’s BNP levels were elevated, and she experienced swelling in her lower extremities. However, following treatment with corticosteroids and immunosuppressive agents, the patient’s chest imaging showed marked improvement, and her overall condition improved significantly, with the resolution of edema, fatigue, and chest tightness.

In this case study, an elderly patient presented with distinct symptoms and signs indicative of heart failure, including chest tightness, facial edema, lower limb edema, and elevated BNP levels. Initially, it was hypothesized that the bilateral pleural effusion was attributable to an excessive cardiac load stemming from hypertension, considering the patient’s medical history of atrial fibrillation and hypertension. Nonetheless, the patient’s blood pressure was well-managed, and following treatment with corticosteroids and immunosuppressants, the pleural effusion was rapidly resolved, and her heart failure symptoms showed improvement. A retrospective study conducted at a single center in Turkey^[7]^ identified that patients with MPA are more likely to exhibit honeycomb lung, atelectasis, interstitial pneumonia, pulmonary venous congestion, and pleural effusion. Furthermore, it has been documented^[8]^ that individuals who are MPO-ANCA-positive have a higher propensity to develop usual interstitial pneumonitis, bronchiectasis, alveolar hemorrhage, pleural effusion, lymph node enlargement, and pulmonary venous congestion. Both studies suggest that pleural effusion is a nonspecific thoracic imaging manifestation in patients with AAV, potentially resulting from systemic inflammation, heart failure, or renal dysfunction. Regarding the propensity for pulmonary venous congestion in MPA patients, we hypothesize that this condition may arise from MPA-induced inflammatory involvement of small pulmonary venules, leading to vascular wall injury, luminal narrowing, and subsequent venous congestion. Alternatively, pulmonary venous congestion could occur secondary to left ventricular failure. In light of the clinical progression observed in our case, we propose that MPA-mediated left ventricular dysfunction precipitated pulmonary venous congestion, thereby contributing to the development of bilateral pleural effusion and pulmonary edema. The patient’s echocardiogram revealed enlargement of both atria, moderate regurgitation of the mitral and tricuspid valves, mild to moderate regurgitation of the aortic valve. If heart failure in a patient is caused by lung disease, the patient will develop pulmonary hypertension, which primarily leads to increased right ventricular afterload. It can present as right ventricular hypertrophy and right ventricular enlargement on echocardiography. However, no pulmonary hypertension or right ventricular abnormalities were found on echocardiography in this patient. Based on the result of the patient’s echocardiogram, we speculate that the enlargement of both atria is caused by the increased volume load due to valvular lesions. Valvular heart disease can be caused by endocarditis. There is a literature report that endocarditis is an uncommon manifestation of AAV, and endocarditis induced by AAV can present as an infrequent type of valvular dysfunction.^[9]^ Therefore, we infer that MPA causes endocarditis, which leads to valve insufficiency and ultimately results in heart failure.

Regrettably, the patient was unable to undergo a lung biopsy due to her advanced age and overall poor health status, which rendered the procedure considerably high-risk. The diagnosis of MPA was established based on clinical manifestations, auxiliary examinations, and the observed efficacy of pharmacological treatment. This case underscores the varied presentations of vasculitis and highlights that a diagnosis should not be dismissed solely on the basis of the absence of renal involvement. Clinicians should remain vigilant for the possibility of MPA in patients exhibiting interstitial pneumonia, recurrent heart failure, bilateral pleural effusion, positive MPO-ANCA, and inadequate responses to anti-heart failure or anti-infective therapies, and should consider initiating appropriate treatment for vasculitis in such cases.

## Author contributions

**Conceptualization:** Jing Chen.

**Formal analysis:** Lvjun Zhang.

**Investigation:** Zhengming Huang, Dan Zhu.

**Resources:** Jing Chen.

**Supervision:** Dan Zhu.

**Visualization:** Lvjun Zhang, Zhengming Huang.

**Writing – original draft:** Jing Chen, Lvjun Zhang.

**Writing – review & editing:** Dan Zhu.
